# A conserved conformation-dependent mechanism for the TREK two-pore domain potassium channel inhibitors carvedilol and fluphenazine

**DOI:** 10.1016/j.molpha.2026.100155

**Published:** 2026-09-12

**Authors:** Joelle Reich, Paul M. Riegelhaupt, Stephen J. Tucker, Conor McClenaghan

**Affiliations:** 1 Center for Advanced Biotechnology and Medicine, Robert Wood Johnson Medical School, Rutgers, the State University of New Jersey, Piscataway, New Jersey; 2 Department of Pharmacology, Robert Wood Johnson Medical School, Rutgers, the State University of New Jersey, Piscataway, New Jersey; 3 Department of Medicine, Robert Wood Johnson Medical School, Rutgers, the State University of New Jersey, Piscataway, New Jersey; 4 Department of Anesthesiology, Weill Cornell Medical College, New York, New York; 5 Clarendon Laboratory, Department of Physics, University of Oxford, Oxford, United Kingdom; 6 Kavli Institute for Nanoscience Discovery, University of Oxford, Oxford, United Kingdom

**Keywords:** TREK-1, TREK-2, Carvedilol, Fluoxetine, Norfluoxetine, Fluphenazine

## Abstract

The TREK subfamily of 2-pore domain potassium channels act as polymodal sensors of multiple physical and chemical stimuli, coupling electrical excitability to the cellular environment. A nuanced understanding of TREK channel pharmacology is necessary for the development of novel experimental tools, for potential pharmacotherapies, and for understanding potential beneficial or adverse effects of clinically used drugs. Here, we sought to determine the mechanism of action of 2 TREK channel inhibitors: the *β*-blocker carvedilol and the antipsychotic fluphenazine. Using patch and 2-electrode voltage-clamp electrophysiology, we show that both carvedilol and fluphenazine exhibit an activation-mode dependence, with mechanical- and temperature-activation of TREK channels reducing drug sensitivity approximately 10-fold. Mutagenesis at a key intramembrane fenestration site leucine, modulator competition experiments, and in silico docking studies are all consistent with carvedilol and fluphenazine binding at a shared, overlapping binding site. These findings establish a conserved conformation-dependent mechanism of action for structurally diverse TREK inhibitors and identify the fenestration as a promiscuous binding site for a range of clinically used drugs.

**Significance Statement:**

TREK potassium channels play key roles in regulating membrane excitability in a wide range of cells. Modulation of these channels has been proposed for potential antiarrhythmic and antidepressant effects. This study defined the off-target molecular mechanism of action of the clinically used drugs carvedilol and fluphenazine on TREK channels and identify the TREK channel fenestration as a promiscuous binding site for inhibitory drugs.

## 1 Introduction

The TREK subfamily of 2-domain potassium (K2P) channels, consisting of TREK-1 (K_2P_2.1; *KCNK2*), TREK-2 (K_2P_10.1; *KCNK10*), and TRAAK (K_2P_4.1; *KCNK4*), is regulated by a wide range of physical and chemical stimuli, including intracellular and extracellular pH, temperature, membrane tension, polyunsaturated fatty acids, and signaling downstream of G protein–coupled receptors.^1^^,^^2^ They are widely expressed in the central and peripheral nervous system where their activity regulates neuronal excitability, and they play key roles in somatosensation, nociception, and temperature sensation.^3^, ^4^, ^5^, ^6^ TREK channel activators are in development as anesthetic or antinociceptive candidates, and inhibitors have been proposed as potential antidepressants or antiarrhythmic molecules.^7^, ^8^, ^9^, ^10^, ^11^, ^12^

TREK channels form as dimers that assemble as either homodimers or heterodimers, with distinct functional properties.^13^, ^14^, ^15^ X-ray crystallographic studies have demonstrated that TREK channels exhibit characteristic K2P channel features including an extracellular cap domain, a pseudo 4-fold symmetrical selectivity filter (SF), and 4 transmembrane helices per monomer that arrange to form intramembrane "fenestrations" (windows within the pore-lining helices that are open to the lipid tails of the membrane) in certain conformations.^16^, ^17^, ^18^, ^19^, ^20^ Channel conformations resolved to date can be categorized into 2 groups: “down-state” conformations, where transmembrane helix 4 (M4) passes the membrane perpendicularly and in which the fenestrations are present, and “up-state” structures, where an upward translocation of M4 to an oblique angle closes the fenestrations. However, gating of the channel primarily occurs at the SF (the “C-type” or “SF-gate”), where the conductive conformation of this gate is promoted by conversion from the down to up conformations in response to mechanical and temperature cues.^19^^,^^21^, ^22^, ^23^, ^24^, ^25^, ^26^, ^27^

Currently, there are no clinically used pharmaceuticals that act selectively on TREK channels, though a wide range of drugs have been demonstrated to modulate the channels in experimental studies.^28^^,^^29^ This diverse range of TREK inhibitors includes multiple drug classes, including the antidepressant fluoxetine and its active metabolite norfluoxetine, the antipsychotic fluphenazine, and the *β*-blocker carvedilol.^19^^,^^23^^,^^30^, ^31^, ^32^, ^33^, ^34^, ^35^, ^36^ Although TREK inhibition is not the primary mechanism of action of these molecules, it is possible that TREK modulation might contribute to either the therapeutic efficacy and/or the adverse clinical effects of these drugs. Recent work has also shown that up/down transitions of M4 can be dynamically controlled by UV light to regulate the SF-gate and induce analgesia via activation of TRAAK in rodent models.^37^

Multiple distinct sites can be targeted by TREK channel inhibitors. At the extracellular face of the channel, ruthenium red potently inhibits TREK-2 (but not TREK-1) via specific interactions within the ion exit pathway at the base of the extracellular cap,^38^ where it acts as a steric blocker of ion conductance.^39^ Access to this site is unaltered in the M4 up/down conformations. By contrast, (nor)fluoxetine binds within the intramembrane fenestrations in a conformational state–specific manner.^19^^,^^23^^,^^31^ Further, recent studies have demonstrated that other TREK/TRAAK modulators such as cannabidiol and the neuroprotective agent sipatrigine bind within the fenestration site, suggesting this is a promiscuous region for drug interactions.^40^^,^^41^ State-dependent effects have also recently been reported for other K2P channel inhibitors such as diacylglycerol, which inhibits TASK channels in a highly state-dependent manner—though its conformational dependence is not as clear as the difference in the fenestration binding site for norfluoxetine in the up versus down conformations of TREK channels.^42^

Understanding the molecular mechanisms of TREK modulators may allow for future development of more potent and selective modulators and assist in providing insights into off-target effects of clinically used drugs. Here, we sought to determine the mechanism by which the clinically used drugs carvedilol and fluphenazine inhibit both TREK-1 and TREK-2 subfamily members.

## 2 Materials and methods

### 2.1 Molecular biology and oocyte and HEK293 cell expression

*Xenopus laevis* oocytes were prepared by collagenase digestion in Ca^2+^-free defolliculation buffer (in mM: 83 NaCl, 2 KCl, 1 MgCl_2_, and 10 HEPES; pH 7.6) supplemented with 2 mg/mL collagenase (type A; Roche), followed by manual defolliculation in enzyme-free buffer. Stage V and VI oocytes were stored in ND96 solution (in mM: 96 NaCl, 2 KCl, 1.8 CaCl_2_, 1 MgCl_2_, and 10 HEPES, 2.5 mM sodium pyruvate, 50 *μ*g/mL gentamycin, 50 *μ*g/mL tetracycline, 50 *μ*g/mL ciprofloxacin, and 100 *μ*g/mL amikacin; pH 7.4). Human TREK-2 (full-length isoform 3; NP_612191) and TASK-1 (NP_002237) in the pBF vector were used for RNA synthesis using the Amplicap SP6 Kit (Cambio). Cells were injected with 1–4 ng RNA up to 4 days after isolation. Experiments were performed 12–24 hours after injection at room temperature (22 °C unless otherwise indicated). Studies were conducted in accordance with project and personal licenses of the Animals (Scientific Procedures) Act 1986 and after approval by the University of Oxford’s local ethical review process.

“Landing Pad” HEK293 cells were cultured in Dulbecco’s modified Eagle’s medium supplemented with 10% FBS and 1% penicillin-streptomycin.^43^^,^^44^ Wild-type (WT) and mutant human TREK-1 and TREK-2 were cloned into an attB vector, which also encoded mCherry and a puromycin-resistance gene, which was cotransfected into the Landing Pad cells alongside a plasmid encoding the Bxb1 integrase for genomic integration of the attB plasmid to generate stable cell lines.^43^^,^^44^ Experiments were performed at least 96 hours after transfection and at room temperature (22 °C unless otherwise indicated). HEK293 cell cultures were not subjected to mycoplasma tests.

### 2.2 Electrophysiology and analysis

Inside-out patch-clamp recordings were made using giant patch electrodes pulled from thick-walled borosilicate glass (pipette resistances around 0.4–0.8 M*Ω* when filled with patch solution). The pipette solution contained (in mM) 116 NaCl, 4 KCl, 1 MgCl_2_, 1.8 CaCl_2_, and 10 HEPES, pH 7.4, whereas the bath solution contained (in mM) 120 KCl, 1 NaCl, 2 EGTA, and 10 HEPES, pH 7.3. The bath solution was exchanged via a gravity flow perfusion system. Data were acquired with pCLAMP and recorded using an Axopatch 200B (Molecular Devices), filtered at 1 kHz, and sampled at 10 kHz. Currents from 0 mV steps were reported unless stated otherwise.

Whole-cell recordings of *Xenopus* oocytes were made by 2-electrode voltage clamp using electrodes pulled from thick-walled borosilicate glass and filled with 3 M KCl. ND96 bath solution was used for all recordings. Data were acquired with pCLAMP and recorded using a GeneClamp 500 amplifier (Molecular Devices). Temperature was controlled via an in-line perfusion heating controller (MultiChannel Systems). Whole-cell patch-clamp recordings of Landing Pad HEK293 cells were made using patch pipettes pulled to tip resistances between 3 and 4.5 M*Ω* when filled with intracellular solution containing (in mM) 140 KCl, 10 NaCl, 1 MgCl_2_, 10 HEPES, 0.5 CaCl_2_, 4 K_2_HPO_4_, and 5 EGTA (pH 7.2 with KOH). The bath solution contained (in mM) 136 NaCl, 6 KCl, 2 CaCl_2_, 1 MgCl_2_, 10 HEPES, and 10 glucose (pH 7.4 with NaOH).

Percentage inhibition was calculated from stable basal currents after excision as follows: % inhibition = (1 − (I_inh_/I_max_)) × 100, where I_inh_ is the current recorded in drug and I_max_ is the maximal current observed. Mechanoactivation was induced by application of 11 mm Hg of negative pressure via the patch pipette manually using a 1-mL syringe (calibrated using a Druck DPI260 pressure indicator). Fold activation = I_activated_/I_basal_, where I_activated_ is the current after application of negative pressure. For quantification of stretch activation, only patches with basal currents >100 pA (at 0 mV) were used. All data are presented as mean ± SD as described. IC50 values were obtained from concentration-response experiments via fitting data using the 4-parameter Hill equation:

(1) $$\text{Normalized}\;\text{current}=I_{\max}/\left(1+{\left(\left[\text{drug}\right]/\text{IC}50\right)}^{h}\right)$$

where I_max_ is the maximal current observed in an experiment, [drug] is the concentration of inhibitor, IC50 is the concentration of half-maximal inhibition, and h is the Hill coefficient. Concentration-response curves were converted to % inhibition for display.

For these exploratory studies, data were subjected to Shapiro-Wilk tests for normal distribution, followed by 1-way ANOVA and post hoc Dunnett’s test or nonparametric Kruskal-Wallis and post hoc Dunn’s tests, or Welch’s *t* test or nonparametric Mann-Whitney *U* test, as described. Statistical tests were conducted in GraphPad Prism 10.5 (GraphPad Software, Inc), and P values <.05 were considered statistically significant.

### 2.3 In silico docking

Blind docking of carvedilol and fluphenazine was performed using Autodock Vina (Scripps Research).^45^ The norfluoxetine-bound TREK-2 structure (protein data bank 4XDK) was prepared for docking by removing norfluoxetine in PyMol (Schrödinger), and adding polar hydrogens, merging nonpolar hydrogens and adding Kollman charges in AutoDockTools.^46^ Carvedilol and fluphenazine 3-dimensional structures were sourced from Pubchem and prepared for docking by ensuring that polar hydrogens were present, nonpolar hydrogens were merged, and Gasteiger charges were added in AutoDockTools (Scripps Research). The top 9 scored binding poses were reported as default and shown for illustration.

## 3 Results

Previously, we demonstrated that norfluoxetine inhibition of TREK-2 was reduced by mechanical and temperature activation.^19^^,^^23^ To determine whether a similar behavior was observed for carvedilol and fluphenazine, we administered both drugs to TREK-2 channels in inside-out voltage-clamp recordings of giant patches excised from *Xenopus* oocytes. In basal conditions (ie, in the absence of additional pressure to the patch), carvedilol inhibited TREK-2 channel activity with an IC50 of ∼7 *μ*M (Fig. 1, A, B, and D). In clear contrast, when channels were activated by the application of negative pressure to the patch pipette, carvedilol sensitivity was markedly reduced (Fig. 1, C and D). Similar results were observed for fluphenazine (Fig. 1, E–H). In contrast, inhibition of TREK-2 by the quaternary ammonium ion tetrapentylammonium, which is predicted to block TREK channels by binding directly below the SF in a non–conformation-dependent manner,^47^^,^^48^ was not altered by mechanoactivation (Fig. 1, I–L).

**Fig. 1:**
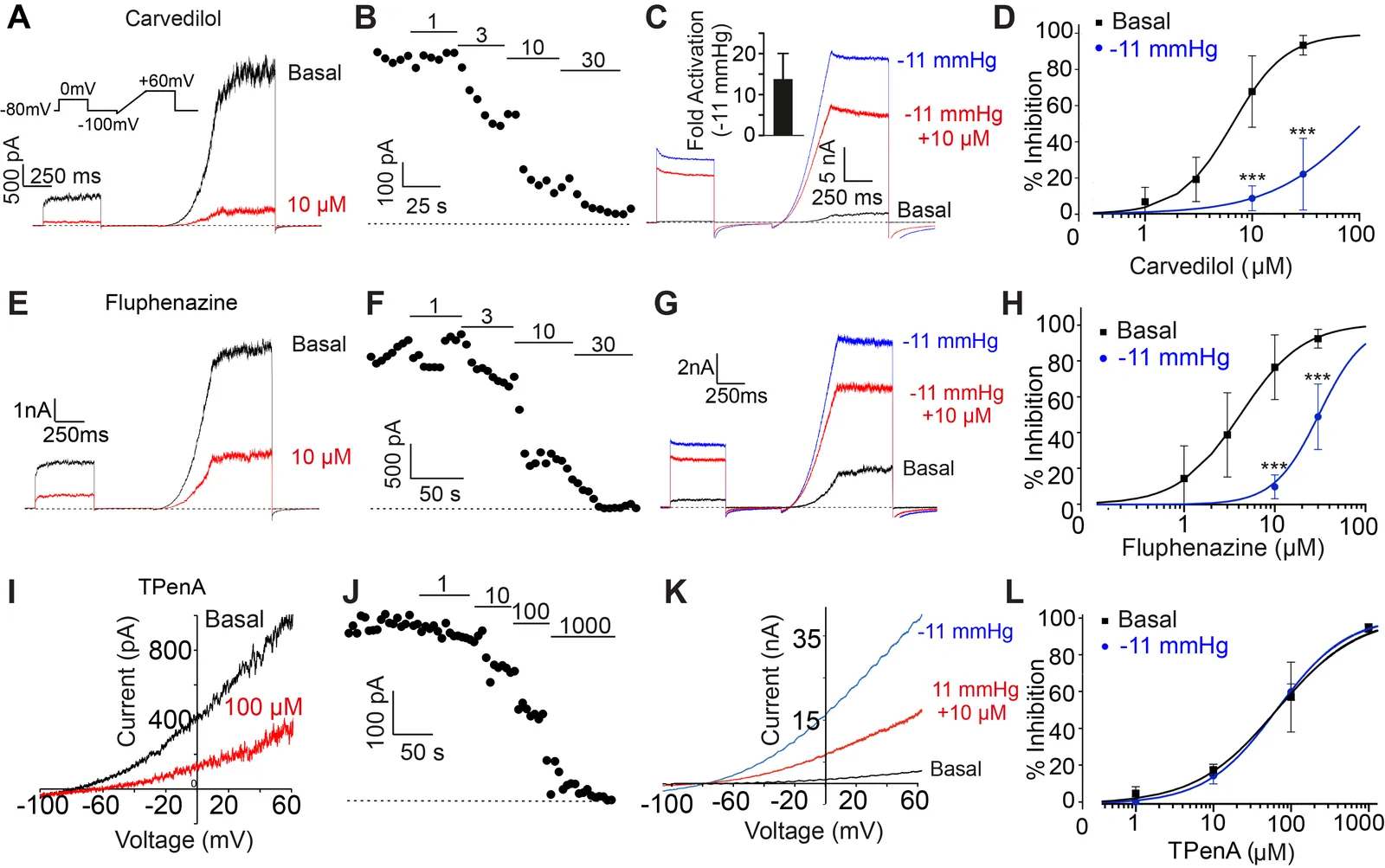
Reduced carvedilol and fluphenazine inhibition of mechanoactivated TREK-2. (A) Example traces showing carvedilol inhibition of basal TREK-2 activity in inside-out giant patch recordings. Black trace: basal current; red trace: current with 10 *μ*M carvedilol. Voltage protocol shown in the inset was applied every 5 seconds in this and all excised patch recordings from oocytes (unless otherwise stated). (B) Time course of an example carvedilol concentration-response experiment showing inhibition of basal TREK-2 activity (concentration in *μ*M indicated; current at 0 mV plotted). (C) Example trace of carvedilol inhibition of mechanoactivated TREK-2 currents. Black trace: basal current; blue trace: current activated by application of −11 mm Hg pressure to the patch pipette; red trace: effect of 10 *μ*M carvedilol on mechanoactivated current. Inset: quantification of fold change in current on application of negative pressure (*n* = 17). (D) Summary carvedilol concentration response for TREK-2 inhibition of basal and mechanoactivated currents in excised patches (*n* = 8 for basal concentration response; *n* = 7 for 10 *μ*M carvedilol with mechanoactivation; and *n* = 5 for 30 *μ*M carvedilol with mechanoactivation). (E) Example traces showing fluphenazine inhibition of basal TREK-2 channel activity in inside-out giant patch recordings. Black trace: basal current; red trace: current in 10 *μ*M fluphenazine. (F) Time course of an example fluphenazine concentration-response experiment showing inhibition of basal TREK-2 activity (concentration in *μ*M indicated; current at 0 mV plotted). (G) Example trace of fluphenazine inhibition of mechanoactivated TREK-2 currents. Black trace: basal current; blue trace: current activated by application of −11 mm Hg suction to the patch pipette; red trace: effect of 10 *μ*M fluphenazine on mechanoactivated current. (H) Summary fluphenazine concentration response for TREK-2 inhibition of basal and mechanoactivated currents in excised patches (*n* = 6 for basal concentration response; *n* = 8 for 10 *μ*M fluphenazine with mechanoactivation; and *n* = 5 for 30 *μ*M fluphenazine with mechanoactivation). (I) Example traces showing TPenA inhibition of basal TREK-2 channel activity in inside-out giant patch recordings. Black trace: basal current; red trace: current in 100 *μ*M TPenA. Currents recorded during 500-millisecond voltage ramps from −100 to 60 mV, repeated every 5 seconds from a holding potential of −80 mV. (J) Time course of an example TPenA concentration-response experiment showing inhibition of basal TREK-2 activity (concentration in *μ*M indicated; current at 0 mV plotted). (K) Example traces showing TPenA inhibition of mechanoactivated TREK-2 currents. Black trace: basal current; blue trace: current activated by application of −11 mm Hg suction to the patch pipette; red trace: effect of 10 *μ*M TPenA on mechanoactivated current. (L) Summary TPenA concentration response for TREK-2 inhibition of basal and mechanoactivated currents in excised patches (*n* = 4 basal; *n* = 5 mechanoactivation). All data are shown as mean ± SD. ∗∗∗ denotes *P* < .001 according to ANOVA and the post hoc Tukey test for pairwise comparison of effects of each concentration on basal vs mechanoactivated currents. TPenA, tetrapentylammonium.

TREK channels are strongly activated by increases in temperature within the physiological range in a likely indirect process that requires cellular integrity.^27^^,^^49^^,^^50^ We therefore used whole-cell 2-electrode voltage-clamp recordings of oocytes expressing TREK-2 to investigate the effect of temperature activation on inhibitor sensitivity. As previously reported,^23^^,^^49^ increasing the bath temperature from 22 °C to 37 °C and 42 °C results in robust increases in TREK-2 currents - and markedly reduced carvedilol inhibition (Fig. 2, A–C). To determine that this was a direct effect of temperature-evoked TREK-2 activation, and not a nonspecific physicochemical effect of the elevated temperature, we measured carvedilol inhibition of TASK-1 (K_2P_3.1/*KCNK3*), a non–temperature-activated K2P family member.^50^ TASK-1 exhibited only a modest increase in currents on increasing temperature, consistent with the weak temperature sensitivity previously reported, and increasing temperature had no effect on drug sensitivity (Fig. 2, D and E). As was observed for mechanoactivation, fluphenazine inhibition of TREK-2 was also reduced by temperature activation (Fig. 2, F and G).

**Fig. 2:**
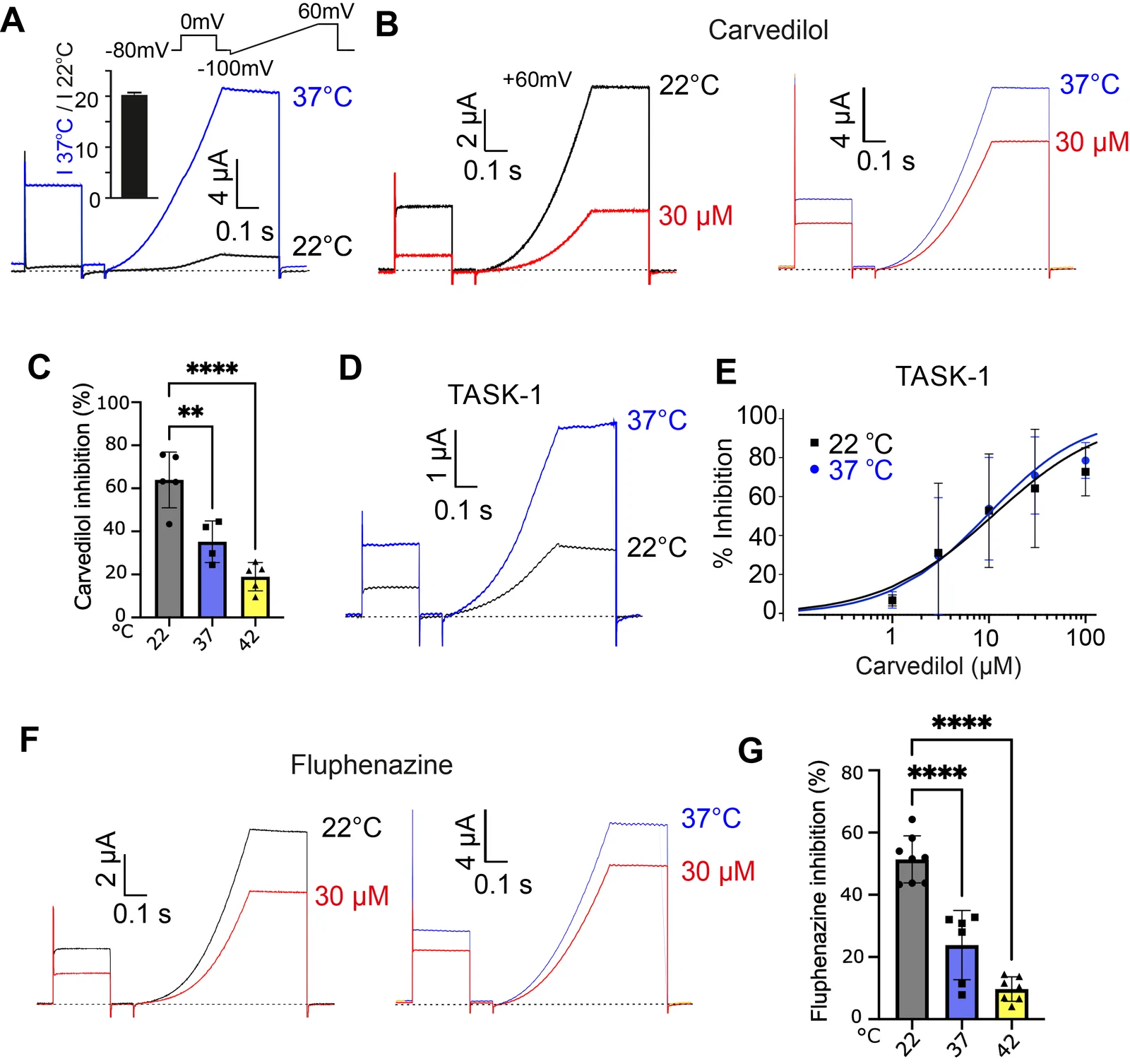
Temperature activation reduces TREK-2 inhibition by carvedilol and fluphenazine. (A) Example traces showing TREK-2 activation by increased bath temperature in 2-electrode voltage-clamp (TEVC) recordings of oocytes. Voltage protocol illustrated was used for all TEVC recordings. The inset shows the increase in current (I) with increasing temperature from basal (22 °C) to 37 °C (*n* = 3). (B) Example traces showing inhibition of basal whole-cell TREK-2 currents (left) or temperature-activated currents at 37 °C (right) by 30 *μ*M carvedilol. (C) Summary percent inhibition by 30 *μ*M carvedilol for TREK-2 currents recorded at 22 °C, 37 °C, and 42 °C (*n* = 5 for 22 °C and 42 °C; *n* = 4 for 37 °C; data shown as mean ± SD). (D) Example trace showing a mild increase in TASK-1 currents with increasing temperature. (E) Summary concentration response for carvedilol for TASK-1 currents recorded at 22 °C or 37 °C (*n* = 8 for 22 °C; *n* = 6 for 37 °C; data shown as mean ± SD). (F) Example traces showing inhibition of basal whole-cell TREK-2 currents (left) or temperature-activated currents at 37 °C (right) by 30 *μ*M fluphenazine. (G) Summary percent inhibition by 30 *μ*M fluphenazine for TREK-2 currents recorded at 22 °C, 37 °C, and 42 °C (*n* = 8 for 22 °C; *n* = 6 for 37 °C; *n* = 7 for 42 °C; data shown as mean ± SD). ∗∗ denotes *P* < .01 and ∗∗∗∗ denotes *P* < .0001 according to ANOVA and the post hoc Tukey test for pairwise comparison of inhibition at different temperatures.

Norfluoxetine binds within the fenestration site near a fenestration lining leucine (L320 in TREK-2), and mutation of this residue to a bulky tryptophan reduces inhibitor sensitivity.^19^^,^^23^ We first sought to test whether this binding site mutation impacts fluphenazine inhibition in TREK-2 and then whether this binding site is conserved in TREK-1, as expected. Importantly, as inhibitor sensitivity is conformation dependent, the results of any mutagenesis study could be complicated by effects of mutations on channel gating. To confirm that the L320W substitution does not influence the equilibrium of channel up and down conformations or affect TREK-2 activity, we measured basal currents in both 2-electrode voltage-clamp recordings from oocytes and whole-cell patch-clamp recordings of Landing Pad HEK293 cell lines expressing WT or TREK-2[L320W]. As shown in Fig. 3, A–D, basal currents and mechanoactivation were essentially unchanged in WT and L320W channels, confirming that this mutation does not have any major effect on channel function. When fluphenazine was administered in whole-cell recordings from Landing Pad HEK293 cells, we observed a marked decrease in fluphenazine potency for TREK-2[L320W] channels, compared with WT channels, indicative of a fenestration binding site for this inhibitor (Fig. 3, E–G).

**Fig. 3:**
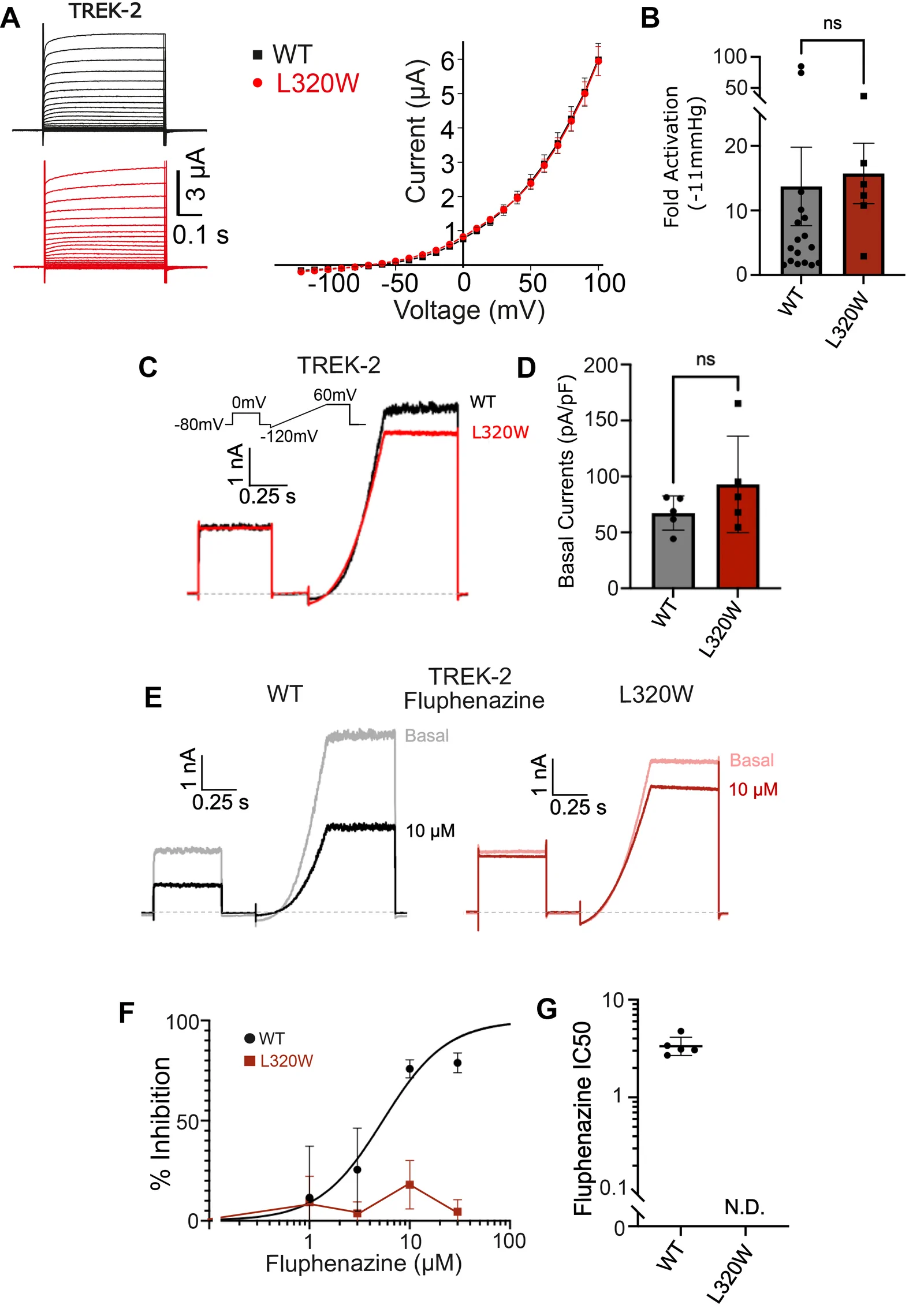
Reduced fluphenazine sensitivity in mutant L320W TREK-2 channels. (A) Basal whole-cell WT TREK-2 and L320W currents recorded from 2-electrode voltage-clamp recordings in *Xenopus* oocytes. Currents were recorded from 500-millisecond voltage steps between −120 and 100 mV at 10 mV increments, holding potential −80 mV. (B) Fold change in current recorded at 0 mV in excised patches from oocytes expressing WT or L320W TREK-2 on application of −11 mm Hg pressure to the patch pipette (*n* = 17 WT; *n* = 6 L320W). (C) Example traces showing whole-cell WT TREK-2 and L320W currents recorded from whole-cell patch-clamp recordings in HEK293 cells. Black trace: TREK-2 WT basal current; red trace: L320W mutant basal current. Voltage protocol shown in the inset was applied every 5 seconds in this and all HEK293 recordings (unless otherwise stated). (D) Current density of cells expressing TREK-2 WT or L320W mutant channels at 0 mV (*n* = 5 for each data set; mean ± SD shown). (E) Example traces showing inhibition of basal (left) TREK-2 WT or (right) L320W inhibition by 10 *μ*M fluphenazine. (F) Summary concentration response of fluphenazine on TREK-2 WT and L320W channels (data shown as mean ± SD). (G) IC50 of fluphenazine on TREK-2 WT or L320W cells (*n* = 5; data shown as mean ± SD). IC50 not determined (N.D.) for L320W channels due to the lack of effect. ns denotes *P* > .05 according to the Mann-Whitney *t* test (B) or Welch’s *t* test (D) for comparison of WT and L320W channels. WT, wild-type.

Next, to test whether this fenestration binding site is conserved in TREK-1, we administered norfluoxetine to both WT and TREK-1 channels with the equivalent fenestration lining leucine (L304) mutated to tryptophan. As expected, the TREK-1 L304W substitution had no effect on basal currents (Fig. 4, A and B) and reduced norfluoxetine potency (Fig. 4, C–E), as previously seen for TREK-2, confirming a conserved binding site for these 2 subfamily members. Fluphenazine also exhibited decreased potency in mutant TREK-1[L304W] channels (Fig. 4, F–H).

**Fig. 4:**
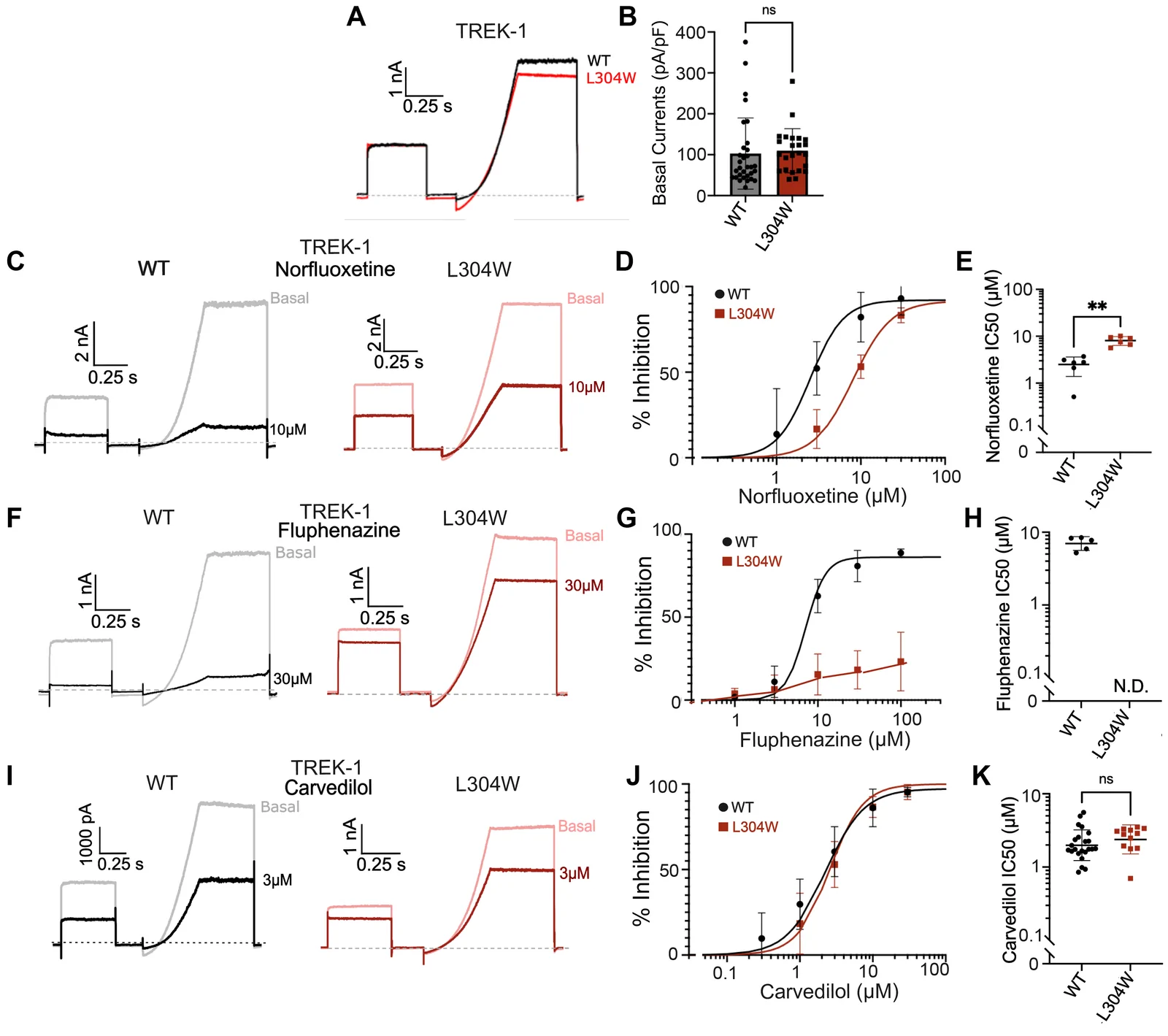
The TREK-1[L304W] substitution reduces norfluoxetine and fluphenazine but not carvedilol sensitivity. (A) Example traces showing basal currents of TREK-1 WT and L304W recorded from whole-cell patch-clamp recordings in HEK293 cells. Black trace: TREK-1 WT; red trace: TREK-1 L304W. (B) Current density of cells expressing WT and L304W mutant TREK-1 channels (*n* = 31 WT; *n* = 24 L304W; data shown as mean ± SD). (C) Example traces showing inhibition of (left) TREK-1 WT and (right) TREK-1 L304W mutant channels by 10 *μ*M norfluoxetine in HEK293 cells. (D) Summary concentration response of norfluoxetine on TREK-1 WT and L320W channels. (E) Norfluoxetine IC50 for WT and L304W TREK-1 channels (*n* = 6 for each data set; data shown as mean ± SD). (F) Example traces showing inhibition of (left) TREK-1 WT and (right) TREK-1 L304W mutant channels by 30 *μ*M fluphenazine in HEK293 cells. (G) Summary concentration response of fluphenazine on TREK-1 WT and L320W channels. (H) Fluphenazine IC50 for WT and L304W TREK-1 channels (*n* = 5 for each data set; data shown as mean ± SD). (I) Example traces showing inhibition of (left) TREK-1 WT and (right) TREK-1 L304W mutant channels by 3 *μ*M carvedilol in HEK293 cells. (J) Summary concentration response of carvedilol on TREK-1 WT and L320W channels. (K) Carvedilol IC50 for TREK-1 WT or L320W channels (*n* = 23 WT; *n* = 12 L304W; data shown as mean ± SD). ∗∗ denotes *P* < .01 and ns denotes *P* > .05 according to the Mann-Whitney test (B), the Kruskal-Wallis test with post hoc Dunn’s multiple comparison test (K), or Welch’s *t* test (E,H) for comparison of WT and L304W channels. WT, wild-type.

Interestingly, in contrast, there was no statistically significant effect of the TREK-1[L304W] mutation on carvedilol inhibition (Fig. 4, I–K), suggesting that this specific residue is not a major determinant of carvedilol binding. To further test whether carvedilol acts via binding within the fenestration, we performed competition experiments in the presence of TREK modulators known to bind in or near the fenestration site. Crystal structures of TREK-2 in complex with a brominated derivative of the channel activator BL1249 suggests that this molecule binds close to the norfluoxetine site, and substitution of fenestration lining residues reduces BL1249-induced channel activation.^47^^,^^51^ Notably, we found that carvedilol potency was markedly decreased in TREK-1 channels activated by BL1249 (Fig. 5, A, D, and E).

**Fig. 5:**
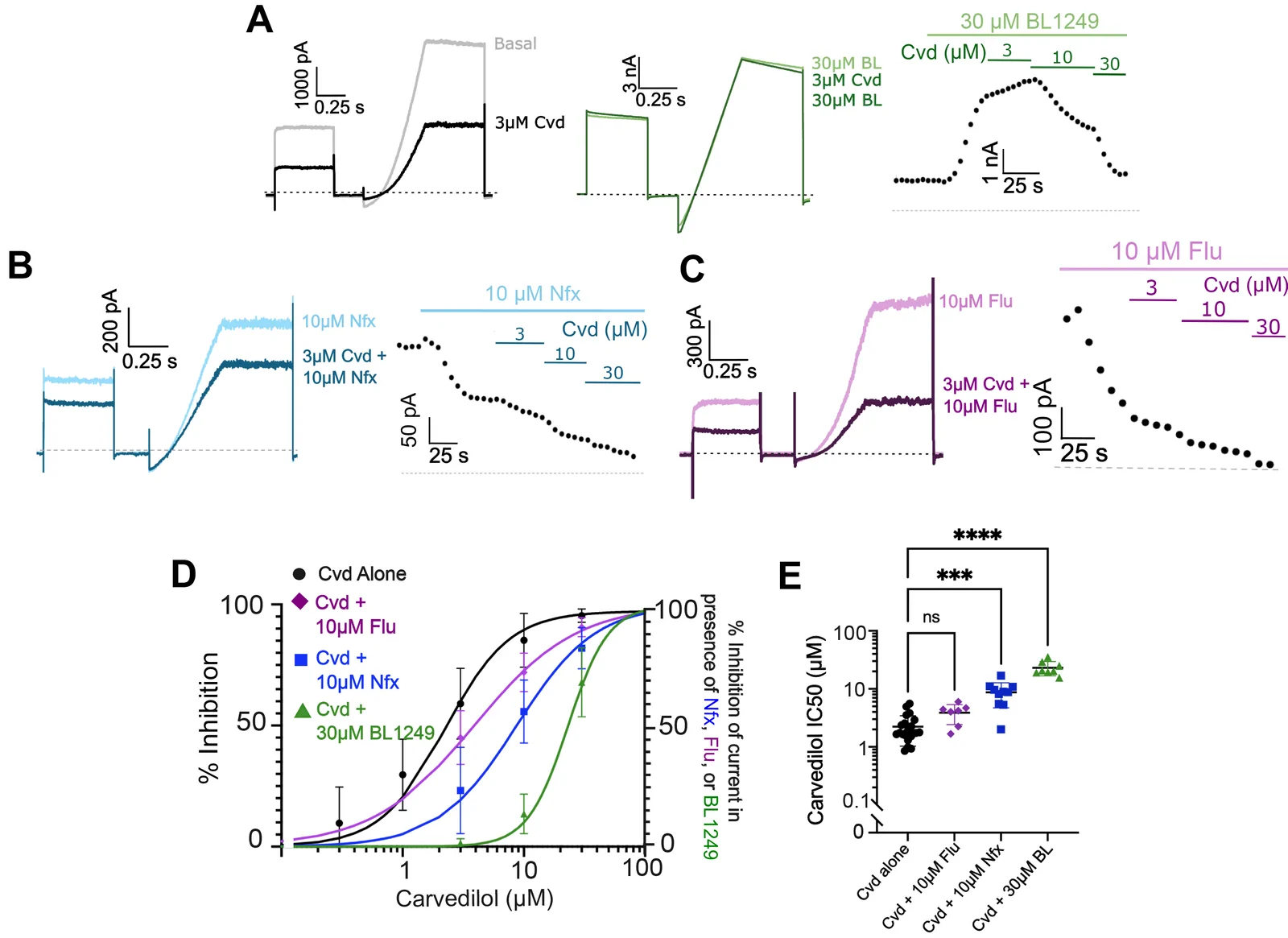
Reduced carvedilol inhibition in the presence of BL1249, norfluoxetine, or fluphenazine. (A) Example traces showing 3 *μ*M carvedilol inhibition of TREK-1 channels expressed in HEK293 cells with (left) no competition or (right) in the presence of 30 *μ*M BL1249. Time course of example BL1249 and carvedilol competition experiment showing activation via BL1249 and inhibition via carvedilol of TREK-1 (current at 0 mV plotted). (B) Example traces showing 3 *μ*M carvedilol inhibition of TREK-1 channels expressed in HEK293 cells in the presence of 10 *μ*M norfluoxetine. Time course of example norfluoxetine and carvedilol competition experiment showing inhibition via norfluoxetine and carvedilol of TREK-1 (current at 0 mV plotted). (C) Example traces showing 3 *μ*M carvedilol inhibition of TREK-1 channels expressed in HEK293 cells in the presence of 10 *μ*M fluphenazine. Time course of example fluphenazine and carvedilol competition experiment showing inhibition via fluphenazine and carvedilol of TREK-1 (current at 0 mV plotted). (D) Summary concentration response of carvedilol on TREK-1 currents in the absence and presence of 10 *μ*M fluphenazine, 10 *μ*M norfluoxetine, or 30 *μ*M BL1249. Carvedilol inhibition normalized to remaining current after fluphenazine or norfluoxetine inhibition. (E) Carvedilol IC50s for TREK-1 channels in the absence and presence of 10 *μ*M fluphenazine, 10 *μ*M norfluoxetine, or 30 *μ*M BL1249 (*n* = 23 for WT; *n* = 7 for fluphenazine; *n* = 10 for norfluoxetine; *n* = 8 for BL1249; data shown as mean ± SD). ∗∗∗ denotes *P* < .001 and ∗∗∗∗ denotes *P* < .0001 according to the Kruskal-Wallis test with post hoc Dunn’s multiple comparison test for comparison of carvedilol in the absence and presence of BL1249, norfluoxetine, or fluphenazine. BL, BL1249; Cvd, carvedilol; Flu, fluphenazine; Nfx, norfluoxetine; ns, not significant; WT, wild-type.

Further, in the presence of 10 *μ*M norfluoxetine, which provokes incomplete inhibition of TREK-1, carvedilol sensitivity was similarly decreased, consistent with norfluoxetine impairing carvedilol binding and suggesting that these drugs compete for an overlapping fenestration binding site (Fig. 5, B, D, and E). However, in the presence of 10 *μ*M fluphenazine, there was only a minor and statistically nonsignificant difference in carvedilol potency (Fig. 5, C–E). This aligns with the finding of reduced potency of fluphenazine but not carvedilol in TREK-1[L304W] variant channels, consistent with these molecules having incompletely overlapping binding sites within the fenestration. Blind in silico docking of both fluphenazine and carvedilol to the TREK-2 down-state crystal structure (protein data bank 4XDK, norfluoxetine removed) produced clusters of binding poses located within the intramembrane fenestrations in close proximity to the crystallographic norfluoxetine binding site (Fig. 6). This, although clearly only a prediction, illustrates that carvedilol and fluphenazine can be accommodated within the fenestration site.

**Fig. 6:**
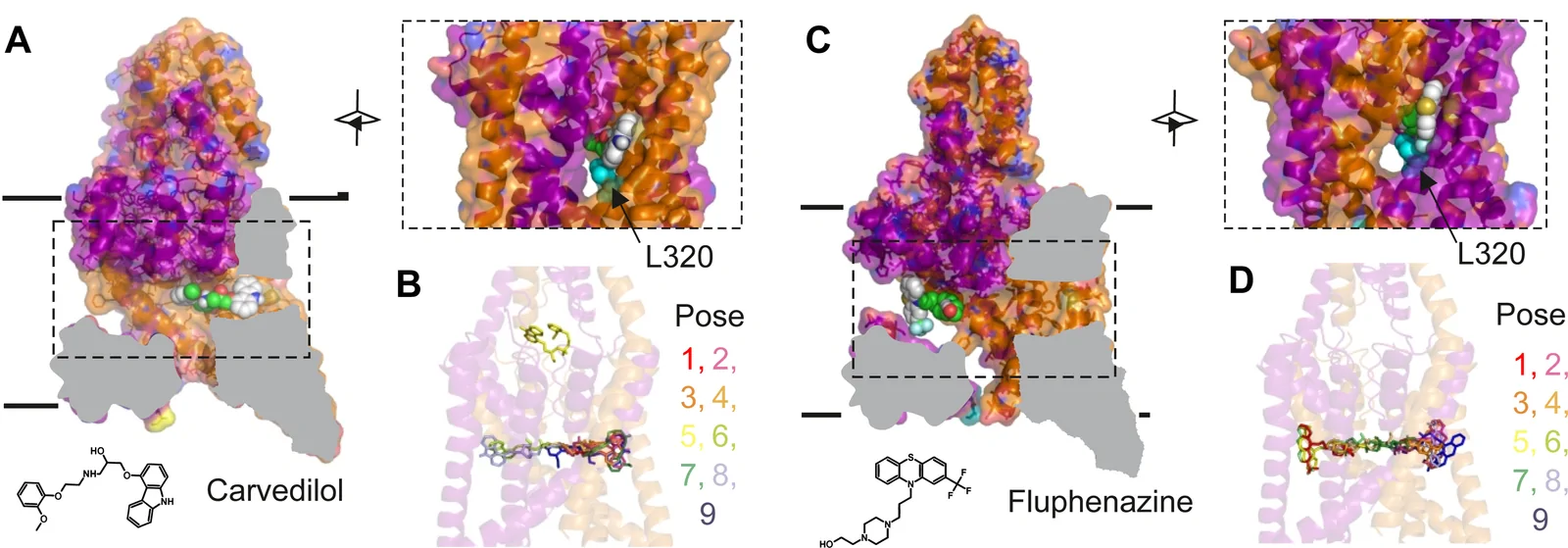
Blind docking predicts potential binding positions for carvedilol and fluphenazine. (A) Representation of the top scored binding pose for carvedilol according to blind docking to TREK-2. Upper right, close-up of carvedilol located within the fenestration. One TREK-2 monomer is in orange, and one in purple. Gray areas represent parts of the dimer that have been removed from view to reveal fenestrations. L320 is highlighted in cyan. Approximate lipid membrane boundaries are represented by black lines. (B) Overlay of top 9 scored binding poses from blind docking showing ensemble of multiple potential poses within the fenestrations (note 1 predicted pose, #5, was suggested above the selectivity filter). (C) Top scoring pose for fluphenazine. (D) Overlay of top scoring poses for fluphenazine. Docking model coordinate files are available in the Supplemental Material.

To further test for a conformation-dependent mechanism of these TREK inhibitors, we also determined drug effects on TREK-1 channels harboring the gain-of-function Y299A substitution. This tyrosine, conserved in TREK-1 and TREK-2, is located on M4 and undergoes different interactions with neighboring residues on M3 when channels interconvert from down to up conformations.^19^ Substitution to alanine provokes increased basal channel activity (Fig. 7, A and B) and blunted stretch activation,^19^ consistent with this mutation promoting the (mechanoactivated) up-state. Stabilization of the up-state is then predicted to decrease access to the fenestration site. In agreement with this model, we found that carvedilol sensitivity is drastically reduced in TREK-1[Y299A] compared with WT channels (Fig. 7, C–E). In contrast, when we tested another activating mutation, TREK-1[W290S], which increases channel open probability by stabilizing the SF gate in the down conformation^20^—leaving the fenestration sites present and accessible—carvedilol sensitivity was not altered (Fig. 7, C–E). Collectively, these results provide evidence that norfluoxetine, carvedilol, and fluphenazine share a conformation-dependent mechanism of action due to a fenestration binding site in TREK channels (Fig. 7F).

**Fig. 7:**
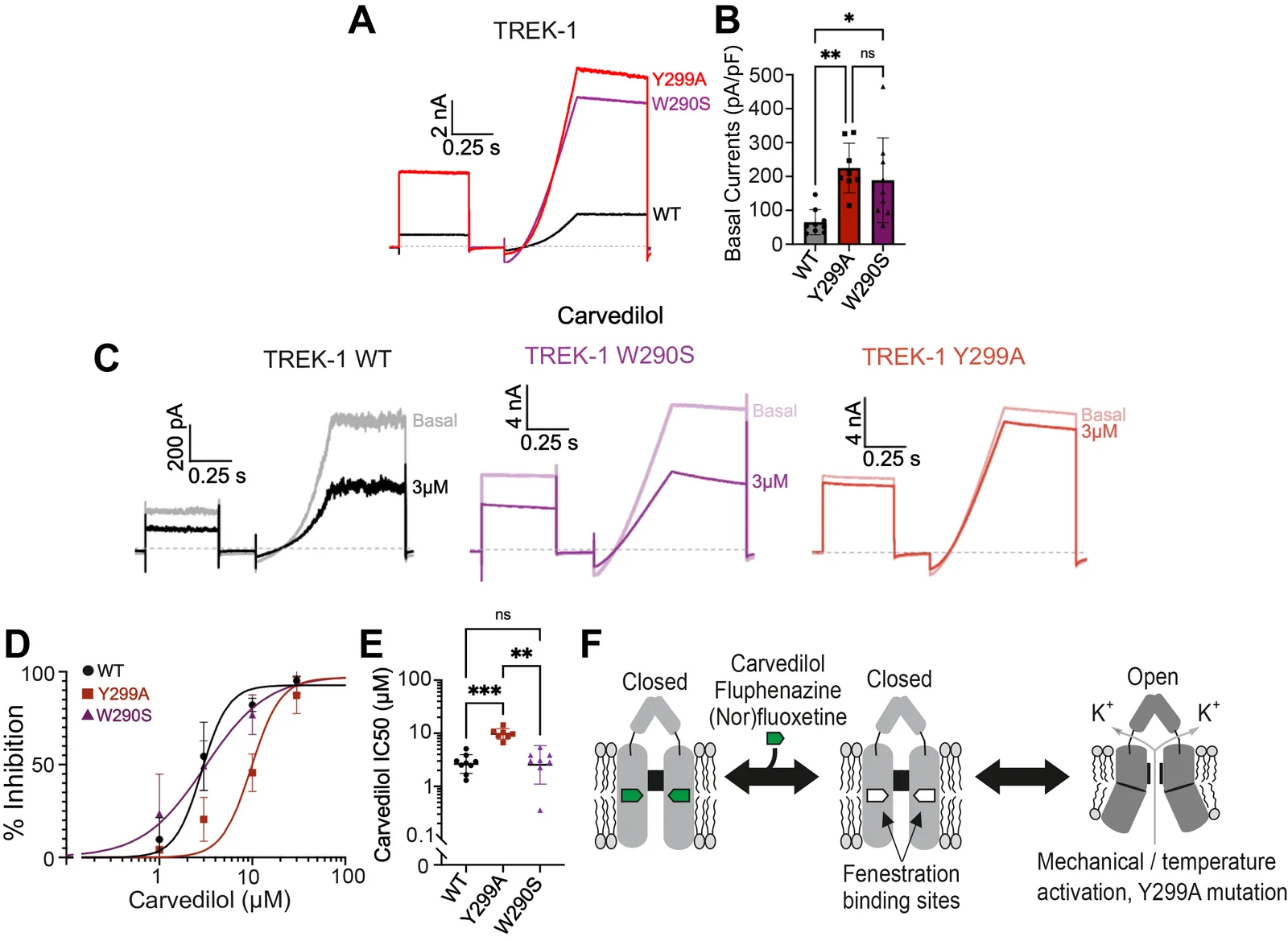
Reduced carvedilol inhibition in TREK-1 Y299A channels compared with WT and mutant W290S channels. (A) Example traces showing basal currents of WT (left), W290S (middle), and Y299A (right) TREK-1 channels from whole-cell patch-clamp recordings of HEK293 cells. (B) Current density of cells expressing WT and Y299A or W290S mutant TREK-1 channels (*n* = 9 for WT and W290S; *n* = 8 for Y299A; data shown as mean ± SD). (C) Example traces showing 3 *μ*M carvedilol inhibition of WT and W290S or Y299A mutant TREK-1 channels. (D) Summary concentration-response curve of carvedilol on WT and Y299A or W290S mutant TREK-1 channels. (E) Carvedilol IC50 for WT and Y299A or W290S mutant TREK-1 channels (*n* = 9 WT, *n* = 7 Y299A, *n* = 8 W290S data shown as mean ± SD). (F) Schematic model for the structural basis of TREK channel inhibition by carvedilol, fluphenazine, and norfluoxetine, which we propose bind to fenestration sites only accessible in the down-state. Mechanical and temperature activation (and activation induced by the TREK-1[Y299A] but not TREK-1[W290S] substitutions) involves conversion to the up-state in which the binding site is abolished. ns denotes *P* > .05, ∗ denotes *P* < .05, ∗∗ denotes *P* < .01, and ∗∗∗ denotes *P* < .001 according to the Kruskal-Wallis test with post hoc Dunn’s multiple comparison test for comparison of carvedilol inhibition or basal current of WT and Y299A or W290S mutant TREK-1 channels. WT, wild-type.

## 4 Discussion

Our data support an emerging model in which the TREK fenestration acts as a promiscuous binding site for a diverse collection of clinically used drugs, which includes inhibitors such as norfluoxetine, cannabidiol, and sipatragine.

Norfluoxetine has previously also been shown to exhibit these conformational state-dependent properties.^19^^,^^23^^,^^31^ The norfluoxetine binding site was shown to be located within intramembrane fenestrations at a site only accessible in “down-state” structures.^19^ This finding provided the basis for studies that demonstrated that mechanoactivation and temperature activation of TREK channels likely involve interconversion from the “down-state” to an “up-state,” via concerted movements of M2, M3, and M4.^23^^,^^24^ Such interconversion abolishes the fenestration binding site, which provides a potential structural explanation of the activation-mode dependence of carvedilol and fluphenazine shown here. Fine mapping of the norfluoxetine site is limited by the moderate resolution of the crystal structure (3.7 Å) and the use of stereoisomers, but the structures suggest that the norfluoxetine aromatic groups may sit within the hydrophobic fenestration,^19^ with the amine group lining the aqueous pore but not obstructing the permeation pathway. Notably, the positive charge of this amine group is not required for inhibition.^51^ Docking experiments predict that carvedilol and fluphenazine may also locate with their respective tricyclic aromatic carbazole and phenothiazine groups stabilized by hydrophobic interactions within the fenestration, but, in contrast to norfluoxetine, the longer side chains are long enough to project into the central vestibule and could directly block ion permeation. Notably, these inhibitors likely occupy incompletely overlapping sites, because mutation of the fenestration lining leucine (L304 in TREK-1/L320 in TREK-2) dramatically impacts fluphenazine but not carvedilol sensitivity, and there was only a small and statistically nonsignificant reduction in carvedilol sensitivity in competition studies with fluphenazine.

How these drugs ultimately inhibit TREK channel opening remains to be established. TREK channel activation is determined by the SF-gate.^7^^,^^21^^,^^22^^,^^25^ Previously, we showed that norfluoxetine binding can slow mechanoactivation of TREK-2, consistent with the drug preventing interconversion from closed down-state to the open up-state.^19^ However, TREK channels can also open from down conformations.^7^^,^^20^^,^^23^ Binding of the drugs to the down-state in the fenestration site likely impairs SF-gate stability to impair ion conduction. Consistent with this, only 3 of the 4 canonical K^+^ ion binding sites were occupied in the norfluoxetine-bound TREK-2 structure.^19^ Interestingly, the TREK channel activator BL1249 also appears to bind in a site that partially overlaps with the fenestration inhibitor site and allows for stabilization of the open SF-gate.^47^ Notably, both norfluoxetine^23^ and, as shown here, carvedilol are able to inhibit TREK-1[W290S] and TREK-2[W306S] channels, despite the SF-gate stabilizing effects of this mutation. This suggests that either fenestration site binding inhibitors can overcome the increased stability imparted by this mutation or there are additional and/or alternative mechanisms of inhibition beyond disruption of the SF-gate.

These studies used full-length TREK channel constructs, but in vivo TREK-2 and TREK-1 are subject to alternative-translation initiation (ATI), resulting in both full-length and N-terminal truncated proteins.^52^^,^^53^ Interestingly, short-form TREK-1 and TREK-2 exhibit reduced sensitivity to carvedilol.^54^ These findings could be seen to implicate the N-terminus, which is spatially distant from the fenestration site, in drug binding. However, inhibition of ATI-associated short-form TREK-1 by the fenestration binding fluoxetine is also reduced, which points toward an indirect effect of N-terminal truncation on conformation-dependent inhibitors.^19^^,^^32^ Notably, the ATI short-form TREK-1 exhibits altered ion selectivity and reduced open probability,^32^^,^^54^^,^^55^ consistent with this N-terminal truncation exerting long-range allosteric effects, which may also impact drug binding at the fenestration site.

TREK channel inhibition has been proposed as a possible therapeutic strategy to address depression^11^^,^^12^^,^^56^ and cardiac arrhythmias,^54^^,^^57^ whereas multiple antipsychotics have been shown to inhibit TREK channels, leading to the hypothesis that TREK inhibition may contribute to their effects.^35^ Importantly, our data indicate that fluphenazine and carvedilol inhibition of TREK channels might be reduced at physiological temperatures, an important consideration for extrapolation of in vitro studies to potential in vivo effects. Carvedilol is used as an antiarrhythmic, but therapeutic plasma concentrations are unlikely to reach levels required to inhibit TREK channels,^54^ with the therapeutic drug plasma concentration measured at up to approximately 0.15 *μ*M—over an order of magnitude lower than the concentrations required for robust TREK inhibition even at room temperature in our studies.^58^, ^59^, ^60^ Antipsychotics, including fluphenazine, concentrate in the brain,^35^^,^^61^ where levels can reach 20 *μ*M^62^—within the inhibitory range for TREK channels in in vitro experiments performed at room temperature here and elsewhere. Ultimately, in vivo, TREK channel sensitivity to carvedilol, fluphenazine, and norfluoxetine will be critically dependent on the conformational state of the channels.

In conclusion, these results establish a common mechanism of TREK channel inhibition for norfluoxetine, carvedilol, and fluphenazine. Our data are consistent with these drugs binding in a conformational state–specific manner to the down-state within intramembrane fenestrations, which represent accommodating sites for a variety of hydrophobic or amphipathic drugs, and which are altered during mechanoactivation and temperature activation of TREK channels (Fig. 7F).

## Conflict of interest

Conor McClenaghan, Joelle Reich, and Paul M. Riegelhaupt report financial support from the NIH. Stephen J. Tucker declares that he has no conflicts of interest.

## References

[1] Noel J., Sandoz G., Lesage F. (2011). Molecular regulations governing TREK and TRAAK channel functions. Channels (Austin).

[2] Honore E. (2007). The neuronal background K2P channels: focus on TREK1. Nat Rev Neurosci.

[3] Alloui A., Zimmermann K., Mamet J. (2006). TREK-1, a K+ channel involved in polymodal pain perception. EMBO J.

[4] Devilliers M., Busserolles J., Lolignier S. (2013). Activation of TREK-1 by morphine results in analgesia without adverse side effects. Nat Commun.

[5] Noël J., Zimmermann K., Busserolles J. (2009). The mechano-activated K+ channels TRAAK and TREK-1 control both warm and cold perception. EMBO J.

[6] Pereira V., Busserolles J., Christin M. (2014). Role of the TREK2 potassium channel in cold and warm thermosensation and in pain perception. Pain.

[7] Lolicato M., Arrigoni C., Mori T. (2017). K2P2.1 (TREK-1)-activator complexes reveal a cryptic selectivity filter binding site. Nature.

[8] Iwaki Y., Yashiro K., Kokubo M. (2019). Towards a TREK-1/2 (TWIK-related K+ channel 1 and 2) dual activator tool compound: multi-dimensional optimization of BL-1249. Bioorg Med Chem Lett.

[9] Yashiro K., Iwaki Y., Urata H. (2025). Discovery of ONO-2920632 (VU6011887): a highly selective and CNS penetrant TREK-2 (TWIK-related K+ channel 2) preferring activator *in vivo* tool compound. ACS Chem Neurosci.

[10] Mathie A., Veale E.L., Cunningham K.P., Holden R.G., Wright P.D. (2021). Two-pore domain potassium channels as drug targets: anesthesia and beyond. Annu Rev Pharmacol Toxicol.

[11] Borsotto M., Veyssiere J., Moha Ou Maati H., Devader C., Mazella J., Heurteaux C. (2015). Targeting two-pore domain K(+) channels TREK-1 and TASK-3 for the treatment of depression: a new therapeutic concept. Br J Pharmacol.

[12] Djillani A., Pietri M., Mazella J., Heurteaux C., Borsotto M. (2019). Fighting against depression with TREK-1 blockers: past and future. A focus on spadin. Pharmacol Ther.

[13] Blin S., Ben Soussia I., Kim E.J. (2016). Mixing and matching TREK/TRAAK subunits generate heterodimeric K2P channels with unique properties. Proc Natl Acad Sci USA.

[14] Levitz J., Royal P., Comoglio Y. (2016). Heterodimerization within the TREK channel subfamily produces a diverse family of highly regulated potassium channels. Proc Natl Acad Sci USA.

[15] Czirjak G., Enyedi P. (2016). Formation of functional heterodimers by TREK-1 and TREK-2 two-pore domain potassium channel subunits. J Biol Chem.

[16] Brohawn S.G., Campbell E.B., MacKinnon R. (2014). Physical mechanism for gating and mechanosensitivity of the human TRAAK K+ channel. Nature.

[17] Brohawn S.G., Campbell E.B., MacKinnon R. (2013). Domain-swapped chain connectivity and gated membrane access in a Fab-mediated crystal of the human TRAAK K+ channel. Proc Natl Acad Sci USA.

[18] Brohawn S.G., del Marmol J., MacKinnon R. (2012). Crystal structure of the human K2P TRAAK, a lipid– and mechano-sensitive K+ ion channel. Science.

[19] Dong Y.Y., Pike A.C., Mackenzie A. (2015). K2P channel gating mechanisms revealed by structures of TREK-2 and a complex with Prozac. Science.

[20] Lolicato M., Riegelhaupt P.M., Arrigoni C., Clark K.A., Minor D.L. (2014). Transmembrane helix straightening and buckling underlies activation of mechanosensitive and thermosensitive K_2P_ channels. Neuron.

[21] Piechotta P.L., Rapedius M., Stansfeld P.J. (2011). The pore structure and gating mechanism of K2P channels. EMBO J.

[22] Schewe M., Nematian-Ardestani E., Sun H. (2016). A non-canonical voltage-sensing mechanism controls gating in K2P K(+) channels. Cell.

[23] McClenaghan C., Schewe M., Aryal P., Carpenter E.P., Baukrowitz T., Tucker S.J. (2016). Polymodal activation of the TREK-2 K2P channel produces structurally distinct open states. J Gen Physiol.

[24] Aryal P., Jarerattanachat V., Clausen M.V. (2017). Bilayer-mediated structural transitions control mechanosensitivity of the TREK-2 K2P channel. Structure.

[25] Brennecke J.T., de Groot B.L. (2018). Mechanism of mechanosensitive gating of the TREK-2 potassium channel. Biophys J.

[26] Clausen M.V., Jarerattanachat V., Carpenter E.P., Sansom M.S.P., Tucker S.J. (2017). Asymmetric mechanosensitivity in a eukaryotic ion channel. Proc Natl Acad Sci USA.

[27] Cordeiro S., Musinszki M. (2025). Thermosensitivity of TREK K2P channels is controlled by a PKA switch and depends on the microtubular network. Pflugers Arch.

[28] Natale A.M., Deal P.E., Minor D.L. (2021). Structural insights into the mechanisms and pharmacology of K(2P) potassium channels. J Mol Biol.

[29] Decher N., Rinne S., Bedoya M., Gonzalez W., Kiper A.K. (2021). Molecular pharmacology of K(2P) potassium channels. Cell Physiol Biochem.

[30] Heurteaux C., Guy N., Laigle C. (2004). TREK-1, a K+ channel involved in neuroprotection and general anesthesia. EMBO J.

[31] Kennard L.E., Chumbley J.R., Ranatunga K.M., Armstrong S.J., Veale E.L., Mathie A. (2005). Inhibition of the human two-pore domain potassium channel, TREK-1, by fluoxetine and its metabolite norfluoxetine. Br J Pharmacol.

[32] Eckert M., Egenberger B., Doring F., Wischmeyer E. (2011). TREK-1 isoforms generated by alternative translation initiation display different susceptibility to the antidepressant fluoxetine. Neuropharmacology.

[33] Lin D.H., Zhang X.R., Ye D.Q. (2015). The role of the two-pore domain potassium channel TREK-1 in the therapeutic effects of escitalopram in a rat model of poststroke depression. CNS Neurosci Ther.

[34] Patel A.J., Honoré E., Lesage F., Fink M., Romey G., Lazdunski M. (1999). Inhalational anesthetics activate two-pore-domain background K+ channels. Nat Neurosci.

[35] Thummler S., Duprat F., Lazdunski M. (2007). Antipsychotics inhibit TREK but not TRAAK channels. Biochem Biophys Res Commun.

[36] Liu H., Enyeart J.A., Enyeart J.J. (2007). Potent inhibition of native TREK-1 K+ channels by selected dihydropyridine Ca2+ channel antagonists. J Pharmacol Exp Ther.

[37] Bied M., Landra-Willm A., Chassot A.A. (2026). Light-induced analgesia provides a drug-free optical method for pain relief via activation of TRAAK k^+^ channels. Nat Commun.

[38] Braun G., Lengyel M., Enyedi P., Czirjak G. (2015). Differential sensitivity of TREK-1, TREK-2 and TRAAK background potassium channels to the polycationic dye ruthenium red. Br J Pharmacol.

[39] Pope L., Lolicato M., Minor D.L. (2020). Polynuclear ruthenium amines inhibit K_2P_ channels via a "finger in the dam" mechanism. Cell Chem Biol.

[40] Docter T., Sorum B., Deshmane R., Doubravsky C., Brohawn S.G. (Preprint. Posted online December 10, 2024). Cannabinoid inhibition of mechanosensitive K^+^ channels. bioRxiv.

[41] Walsh Y., Leach M., Veale E.L., Mathie A. (2021). Block of TREK and TRESK K2P channels by lamotrigine and two derivatives sipatrigine and CEN-092. Biochem Biophys Rep.

[42] Jouen-Tachoire T.R.H., Proks P., Seiferth D. (2026). Structural determinants for GPCR-mediated inhibition of TASK K2P channels by diacylglycerol and its dysfunction in disease. EMBO J.

[43] Gao J., McClenaghan C., Matreyek K.A., Grange D.K., Nichols C.G. (2023). Rapid Characterization of the functional and pharmacological consequences of Cantú syndrome K_ATP_ channel mutations in intact cells. J Pharmacol Exp Ther.

[44] Matreyek K.A., Stephany J.J., Chiasson M.A., Hasle N., Fowler D.M. (2020). An improved platform for functional assessment of large protein libraries in mammalian cells. Nucleic Acids Res.

[45] Trott O., Olson A.J. (2010). AutoDock Vina: improving the speed and accuracy of docking with a new scoring function, efficient optimization, and multithreading. J Comput Chem.

[46] Morris G.M., Huey R., Lindstrom W. (2009). AutoDock4 and AutoDockTools4: automated docking with selective receptor flexibility. J Comput Chem.

[47] Schewe M., Sun H., Mert Ü. (2019). A pharmacological master key mechanism that unlocks the selectivity filter gate in K^+^ channels. Science.

[48] Lenaeus M.J., Burdette D., Wagner T., Focia P.J., Gross A. (2014). Structures of KcsA in complex with symmetrical quaternary ammonium compounds reveal a hydrophobic binding site. Biochemistry.

[49] Kang D., Choe C., Kim D. (2005). Thermosensitivity of the two-pore domain K+ channels TREK-2 and TRAAK. J Physiol.

[50] Maingret F., Lauritzen I., Patel A.J. (2000). TREK-1 is a heat-activated background K(+) channel. EMBO J.

[51] Proks P., Schewe M., Conrad L.J. (2021). Norfluoxetine inhibits TREK-2 K2P channels by multiple mechanisms including state-independent effects on the selectivity filter gate. J Gen Physiol.

[52] Thomas D., Plant L.D., Wilkens C.M., McCrossan Z.A., Goldstein S.A.N. (2008). Alternative translation initiation in rat brain yields K2P2.1 potassium channels permeable to sodium. Neuron.

[53] Simkin D., Cavanaugh E.J., Kim D. (2008). Control of the single channel conductance of K2P10.1 (TREK-2) by the amino-terminus: role of alternative translation initiation. J Physiol.

[54] Kisselbach J., Seyler C., Schweizer P.A. (2014). Modulation of K2P 2.1 and K2P 10.1 K(+) channel sensitivity to carvedilol by alternative mRNA translation initiation. Br J Pharmacol.

[55] Veale E.L., Al-Moubarak E., Bajaria N. (2014). Influence of the N terminus on the biophysical properties and pharmacology of TREK1 potassium channels. Mol Pharmacol.

[56] Heurteaux C., Lucas G., Guy N. (2006). Deletion of the background potassium channel TREK-1 results in a depression-resistant phenotype. Nat Neurosci.

[57] Li J., Janin A., Patoughi M. (2024). Circulating autoantibodies targeting TREK-1 in patients with short-coupled ventricular fibrillation. Circulation.

[58] Hwang S., Lee S., Yoon J., Chung J.Y. (2023). Population pharmacokinetic-pharmacodynamic modeling of carvedilol to evaluate the effect of cytochrome P450 2D6 genotype on the heart rate reduction. J Korean Med Sci.

[59] Kim Y.H., Choi H.Y., Noh Y.H. (2015). Dose proportionality and pharmacokinetics of carvedilol sustained-release formulation: a single dose-ascending 10-sequence incomplete block study. Drug Des Devel Ther.

[60] Nikolic V.N., Jankovic S.M., Velickovic-Radovanović R. (2013). Population pharmacokinetics of carvedilol in patients with congestive heart failure. J Pharm Sci.

[61] Vivier D., Bennis K., Lesage F., Ducki S. (2016). Perspectives on the two-pore domain potassium channel TREK-1 (TWIK-related K(+) channel 1). A novel therapeutic target?. J Med Chem.

[62] Aravagiri M., Marder S.R., Yuwiler A., Midha K.K., Kula N.S., Baldessarini R.J. (1995). Distribution of fluphenazine and its metabolites in brain regions and other tissues of the rat. Neuropsychopharmacology.

