# Supplementary material for: A conserved conformation-dependent mechanism for the TREK two-pore domain potassium channel inhibitors carvedilol and fluphenazine
Source: Mol Pharmacol. 2026 Sep 12;108(10):100155. doi: 10.1016/j.molpha.2026.100155 (PMC13727974; doi:10.1016/j.molpha.2026.100155)
Supplement: Supplementary file 1 [file mmc1.docx]

**A conserved conformation-dependent mechanism for the TREK K2P channel inhibitors carvedilol and fluphenazine**

Joelle Reich, Paul M Riegelhaupt, Stephen J. Tucker, Conor McClenaghan

**Docking model files:**

**Supplemental PBD 4xdk_for_docking.pdb:** Human TREK-2 crystal structure (PDB: 4xdk) with norfluoxetine manually removed. This structure was used for all subsequent docking.

**CARV_POSE_1 to CARV_POSE_9**: Top scored poses for carvedilol in docking studies using 4xdk_for_docking.pdb.

**FLUP_POSE_1 to FLUP_POSE_9:** Top scored poses for fluphenazine in docking studies using 4xdk_for_docking.pdb.
